# Privacy-, linguistic-, and information-preserving synthesis of clinical documentation through generative agents

**DOI:** 10.3389/frai.2025.1644084

**Published:** 2025-09-16

**Authors:** Mark van Velzen, Robert F. van der Willigen, Vincent J. de Beer, Helen I. de Graaf-Waar, Esther R. C. Janssen, Sjemaine van Leeuwen, Micha F. van der Willigen, Martijn J. van der Willigen, Gavin Renardus, Rayan El Maaroufi, Sven J. Satimin, Larissa M. Hartog, Tim Hulsen, Nico L. U. van Meeteren, Mark C. Scheper

**Affiliations:** ^1^Data Supported Healthcare: Data-Science Unit, Research Center Innovations in Care, Rotterdam University of Applied Sciences, Rotterdam, Netherlands; ^2^Department of Anesthesiology and Department of Cariothoracic Surgery, Erasmus Medical Center, Rotterdam, Netherlands; ^3^HR Datalab EAS, School of Engineering and Applied Science, Rotterdam University of Applied Sciences, Rotterdam, Netherlands,; ^4^School of Communication, Media and Information Technology, Rotterdam University of Applied Sciences, Rotterdam, Netherlands; ^5^Department of Orthopedic Surgery, VieCuri Medical Centre, Venlo, Netherlands; ^6^Radboud Institute for Health Sciences, IQ Health, Radboud University Medical Center, Nijmegen, Netherlands; ^7^School of Allied Health, HAN University of Applied Sciences, Nijmegen, Netherlands; ^8^Medifit Bewegingscentrum, Oss, Netherlands; ^9^Data Science & AI Engineering, Philips, Eindhoven, Netherlands; ^10^Top Sector Life Sciences and Health (Health~Holland), The Hague, Netherlands; ^11^Allied Health Professions, Faculty of Medicine and Science, Macquarrie University, Sydney, NSW, Australia; ^12^Solid Start Coalition, Erasmus Medical Center, Rotterdam, Netherlands

**Keywords:** healthcare, data synthesis, privacy, generative agents, linguistics, information theory, synthetic health data generation (SHDG), clinical natural language processing (NLP)

## Abstract

The widespread adoption of generative agents (GAs) is reshaping the healthcare landscape. Nonetheless, broad utilization is impeded by restricted access to high-quality, interoperable clinical documentation from electronic health records (EHRs) due to persistent legal, ethical, and technical barriers. Synthetic health data generation (SHDG), leveraging pre-trained large language models (LLMs) instantiated as GAs, could offer a practical solution by creating synthetic patient information that mimics genuine EHRs. The use of LLMs, however, is not without issues; significant concerns remain regarding privacy, potential bias propagation, the risk of generating inaccurate or misleading content, and the lack of transparency in how these models make decisions. We therefore propose a privacy-, linguistic-, and information-preserving SHDG protocol that employs multiple context-aware, role-specific GAs. Guided by targeted prompting and authentic EHRs—serving as structural and linguistic templates—role-specific GAs can, in principle, operate collaboratively through multi-turn interactions. We theorized that utilizing GAs in this fashion permits LLMs not only to produce synthetic EHRs that are accurate, consistent, and contextually appropriate, but also to expose the underlying decision-making process. To test this hypothesis, we developed a no-code GA-driven SHDG workflow as a proof of concept, which was implemented within a predefined, multi-layered data science infrastructure (DSI) stack—an integrated ensemble of software and hardware designed to support rapid prototyping and deployment. The DSI stack streamlines implementation for healthcare professionals, improving accessibility, usability, and cybersecurity. To deploy and validate GA-assisted workflows, we implemented a fully automated SHDG evaluation framework—co-developed with GenAI technology—which holistically compares the informational and linguistic features of synthetic, anonymized, and real EHRs at both the document and corpus levels. Our findings highlight that SHDG implemented through GAs offers a scalable, transparent, and reproducible methodology for unlocking the potential of clinical documentation to drive innovation, accelerate research, and advance the development of learning health systems. The source code, synthetic datasets, toolchains and prompts created for this study can be accessed at the GitHub repository: https://github.com/HR-DataLab-Healthcare/RESEARCH_SUPPORT/tree/main/PROJECTS/Generative_Agent_based_Data-Synthesis.

## 1 Introduction

While the use of GenAI in healthcare offers substantial promise and is expected to become an integral part of regular clinical practice, its widespread adoption is limited by several critical challenges (Sai et al., 2024). These include fragmented and often inaccessible data silos, significant variability in the quality of real-world data, and complex ethical and legal considerations. Efforts to address these barriers are underway, including the implementation of federated learning approaches, the establishment of robust data governance frameworks that incorporate standards such as HL7 FHIR and FAIR principles, and the development of evolving regulatory measures—such as the European Union AI Act and the General Data Protection Regulation. Collectively, these initiatives aim to foster a landscape of responsible and ethical innovation in healthcare AI (Mons et al., 2017; Busch et al., 2024; Woisetschläger et al., 2024; Ibrahim et al., 2025; Liu et al., 2025). For readers seeking concrete real-world clinical practice use cases of GenAI in healthcare information systems, see Sai et al. (2024) and Reddy (2024).

Real-world data as recorded in EHRs consists primarily of free-text narratives that often contain valuable clinical insights that are not captured by structured data formats (Negro-Calduch et al., 2021; Seinen et al., 2024). However, the synthesis of meaningful health-related text has received little attention in GenAI literature (Murtaza et al., 2023; Ibrahim et al., 2025; Loni et al., 2025; Rujas et al., 2025). This represents a missed opportunity, not only in terms of data synthesis methodology, but also because free-text narratives often contain hidden and nuanced clinical information that is essential for meaningful patient understanding, accelerated early diagnosis, and improved clinical decision making (Negro-Calduch et al., 2021; Seinen et al., 2024; Schut et al., 2025). Moreover, while methods for synthesizing structured EHR data are well established, the generation of synthetic free text from real-world EHRs remains a comparatively underdeveloped area (Murtaza et al., 2023; Ibrahim et al., 2025; Rujas et al., 2025).

High quality synthetic health data generation (SHDG) that emulates real-world clinical documents is emerging as a vital strategy to enable safe, scalable, and privacy-preserving GenAI implementation in health and care settings (Smolyak et al., 2024; Loni et al., 2025). To be precise, SHDG is a privacy-enhancing technology that entails the generation of synthetic data based on real-world datasets. These synthetic datasets are designed to retain the essential statistical patterns and relationships of the original data, without containing any directly identifiable information. The goal is to enable analyses on synthetic data that produce results closely mirroring those obtained from the real data (Murtaza et al., 2023; Drechsler and Haensch, 2024; Ibrahim et al., 2025; Rujas et al., 2025). Ideally fully interoperable and machine-readable, synthetic datasets provide a foundation for the development, testing, and validation of innovative applications ranging from personalized healthcare models to solutions that alleviate administrative workload (Ibrahim et al., 2025; Rujas et al., 2025).

The growing promise and pitfalls of SHDG is best understood in the context of major breakthroughs in natural language processing (NLP) and GenAI, spanning early statistical approaches to today’s transformer-based language models. Synthetic data generation gained momentum in the early 1990s exemplified by Rubin’s (Rubin, 1993) multiple imputation framework and Little’s efforts on statistical disclosure through data masking (Little, 1993). Since 2010 the adoption of machine learning and deep learning has expanded the applications of synthetic data, especially in healthcare (Eigenschink et al., 2023; Murtaza et al., 2023; Drechsler and Haensch, 2024; Goyal and Mahmoud, 2024; Pezoulas et al., 2024). Deep learning models can automatically extract and represent intricate patterns from large datasets, thereby facilitating more accurate and efficient processing of information. Early approaches used models called convolutional and recurrent neural networks, which allowed machines to automatically process unstructured datasets such as images and speech without the need for feature analysis. However, deep learning model development is labor-intensive and complex (LeCun et al., 2015; Goodfellow et al., 2018).

The use of Generative Adversarial Networks (GANs) has led to significant advances in data synthesis and can be classified as both deep learning and GenAI approaches. GANs rely on adversarial training, in which two deep neural networks—the generator and the discriminator—compete with one another until the discriminator can no longer distinguish between real and synthetic data, reaching equilibrium (Goodfellow et al., 2018; Baowaly et al., 2019; Ibrahim et al., 2025). It is the most widely adopted AI technology in the fields of health and healthcare for generating synthetic data. GAN models were originally developed to produce realistic pictures—such as images from magnetic resonance imaging (MRI) or dermatoscopy—as well as to create continuous data. Continuous data includes numerical values that can take on any value within a range, like blood pressure, glucose levels, or patient age. In addition, specialized GAN models have also been used to generate time-series data, such as signals from electrocardiograms (ECG) and electroencephalograms (EEG) (Ibrahim et al., 2025).

The advent of transformer-based architectures revolutionized NLP by significantly enhancing language understanding (Vaswani et al., 2017). Groundbreaking pre-trained language models such as “bidirectional encoder representations from transformer” (BERT) laid the groundwork for this transition by introducing mechanisms for capturing nuanced contextual relationships within text. Building upon this foundation, domain-specific adaptations like BioBERT and Clinical-BERT, trained on biomedical literature and clinical notes, respectively, substantially increased the fidelity with which deep learning could represent the intricate semantics of medical language through vectorization and embeddings of text into numerical expressions (Alsentzer et al., 2019; Lee et al., 2020; Bommasani et al., 2021; Ibrahim et al., 2025). Capturing clinically meaningful concepts through tokenization, vectorization, and text embedding, however, remains a formidable challenge. For example, clinical notes contain valuable contextual information but are characterized by a variety of nomenclatures, abbreviations, misspellings, and synonyms both within and across healthcare disciplines (Cannon and Lucci, 2010; Doan et al., 2014; Meystre et al., 2017; Negro-Calduch et al., 2021; Seinen et al., 2024; Schut et al., 2025).

The evolution of transformer-based architectures culminated in the development of Large Language Models (LLMs) that are not only capable of language understanding—like BERT—but are also capable of language generation—like OpenAI’s GPT3—, called foundational models (Bommasani et al., 2021). LLMs further amplified generative and NLP capabilities—made possible by scaling up model architectures, leveraging enhanced computational power, and training on extremely large datasets. The emergence of novel functionality is associated with the so-called “scaling effect”—a phenomenon that was initially unforeseen and, to this day, remains inadequately understood (Kaplan et al., 2020; Coveney and Succi, 2025). Recent comprehensive surveys underscore the complexity and ongoing debate surrounding the generality of scaling laws, highlighting both their impressive predictive successes and significant limitations—especially when LLMs are applied in situations quite different from the functionality they were originally trained for, or when they face new types of data they have not seen before (Li et al., 2023; Sengupta et al., 2025). One of the most striking emergent properties of LLMs is their ability to respond to prompting—where users provide specific instructions or examples in natural language to guide the model’s output. This phenomenon allows for highly flexible and tailored use of LLMs without the need for additional fine-tuning, thereby broadening their practical utility in clinical document generation and comprehension. For example, prompting allows for zero-shot and few-shot learning, wherein LLMs can generate appropriate responses to tasks or questions with either no examples (zero-shot) or just a handful of provided examples (few-shot), greatly enhancing their versatility to synthesize realistic clinical narratives (Brown et al., 2020).

The rapid development of efficient, fine-tunable Small Language Models (SLMs) and multimodal SLMs is rapidly transforming healthcare. Unlike their bigger LLM counterparts SLMs can operate on-premises servers supporting local deployment, which significantly reduces costs, carbon footprint, and privacy concerns. These advances—as well as techniques like quantization, which reduce model size and accelerate inference time—are making it feasible to bring powerful language understanding and generation capabilities directly to the point of care, enabling clinical decision support, documentation, and patient interaction without reliance on high-end cloud infrastructure (Schick and Schütze, 2021; Dibia et al., 2024; Garg et al., 2025; Kim et al., 2025; Xie et al., 2025).

However, as GenAI becomes increasingly integrated into clinical workflows, it faces unique challenges specific to the medical domain. One of the most persistent and technically demanding issues is the precise segmentation and representation of multi-word clinical terms—such as “low blood pressure” or “low back pain” as well as their common abbreviations like “LBP.” Language models, including both LLMs and SLMs, often struggle to consistently recognize and encode such terms as unified clinical concepts. Inconsistent tokenization or lack of domain-specific context can lead to fragmented or distorted semantic representations, which in turn may compromise the accuracy of clinical information extraction, decision support, or narrative synthesis. This underscores the ongoing need for the development of more sophisticated prompting strategies tailored for healthcare, and the integration of concept embeddings, clinical practice guidelines, and real-world sample data to improve the realism and utility of synthetic clinical data (Beam et al., 2020; Chung et al., 2023; Han et al., 2023; Li et al., 2023).

## 2 GA-assisted SHDG workflows

To overcome the challenges of semantic fragmentation and context loss, recent research has shifted toward collaborative, multi-agent workflows to jointly tackle the complexity of clinical narratives and multimodal data. This new class of GenAI—known as Agentic AI or Generative Agents (GAs)—features multiple autonomous agents, each specializing in a particular task or sensory domain such as vision, language, audio, or touch (Chan et al., 2023; Park et al., 2023). That is, each agent can preside over its own dedicated LLM, prompted to address the unique challenges of its modality and dedicated task. By collaborating iteratively and sharing insights across these different modalities, GAs are able to process and integrate information from multiple sources—text, images, speech—generating content that is coherent and contextually rich (Qiu et al., 2024; Gridach et al., 2025; Hettiarachchi, 2025; Piccialli et al., 2025; Schneider, 2025).

Unlike traditional GenAI, GAs can gather real-time data from various sources, use different tools, design custom workflows, and refine their strategies through feedback, making them highly flexible and context aware. This collaborative approach allows specialized agents to solve complex tasks—such as SHDG—within a single workflow (Qiu et al., 2024). Some advanced workflows also incorporate language-agnostic concept models, which can further enhance the quality and performance of data synthesis (Daull et al., 2023; Barrault et al., 2024; Xie et al., 2024; Jin et al., 2025).

Building on this flexible and context-aware foundation, role-specific GAs are able to distribute cognitive workloads and leverage the strengths of multiple specialized agents within a single workflow (Xie et al., 2024; Jin et al., 2025). This not only allows for more effective problem solving but also enables the application of domain-specific expertise and enhances system robustness across a range of complex real-world tasks. For example, by leveraging advanced language models such as GPT-4, GAs can support workflows in scientific literature review writing (Abdurahman et al., 2025), sentiment analysis (Vasireddy et al., 2024), and the identification of mental health symptoms such as social anxiety from clinical interview data (Ohse et al., 2024). GAs also demonstrate promise in interpreting and structuring unstructured data from EHRs and clinical documentation (Yang et al., 2025). In particular, GPT-4.1 can complement—and sometimes rival—human expertise in global health education and data analysis (Thandla et al., 2024).

Low-code/no-code LLM platforms, such as Flowise,^1^ Langflow,^2^ and AutoGen^3^ (Kumar, 2023), are transforming the way GA-assisted SHDG workflows could be developed (Jeong, 2025). These platforms use drag-and-drop interfaces and natural language prompts, making it much easier to create, modify, and optimize workflows that leverage LLMs without the requirement of in-depth coding expertise. As a result, healthcare professionals and other non-technical users can now play an active role in designing and improving intelligent systems relevant to their needs. Moreover, a recent development called “Vibe-coding”—coined by Karpathy (2025)—illustrates this shift by enabling conversational co-development. Here, developers interact with GenAI tools—like GitHub Copilot—using plain language to iteratively refine and adjust code (Karpathy, 2025; Mayo, 2025).

In what follows, we present a protocolized GA-driven proof-of-concept for SHDG use cases aimed at creating novel synthetic clinical documents that emulate genuine EHRs while safeguarding patient privacy, preserving linguistic integrity, and maintaining informational accuracy under conditions of limited access to authentic EHR datasets.

## 3 Materials and methods

The protocol described here aims to expand the responsible use and deployment of GenAI healthcare solutions to a broad spectrum of end-users—including those without specialized AI expertise—by providing clear, step-by-step guidance for designing workflows that leverage GAs for SHDG. To further support open-source adoption, reproducibility and practical application of our GA-assisted SHDG workflows we provide a GitHub repository.^4^

**Figure 1:**
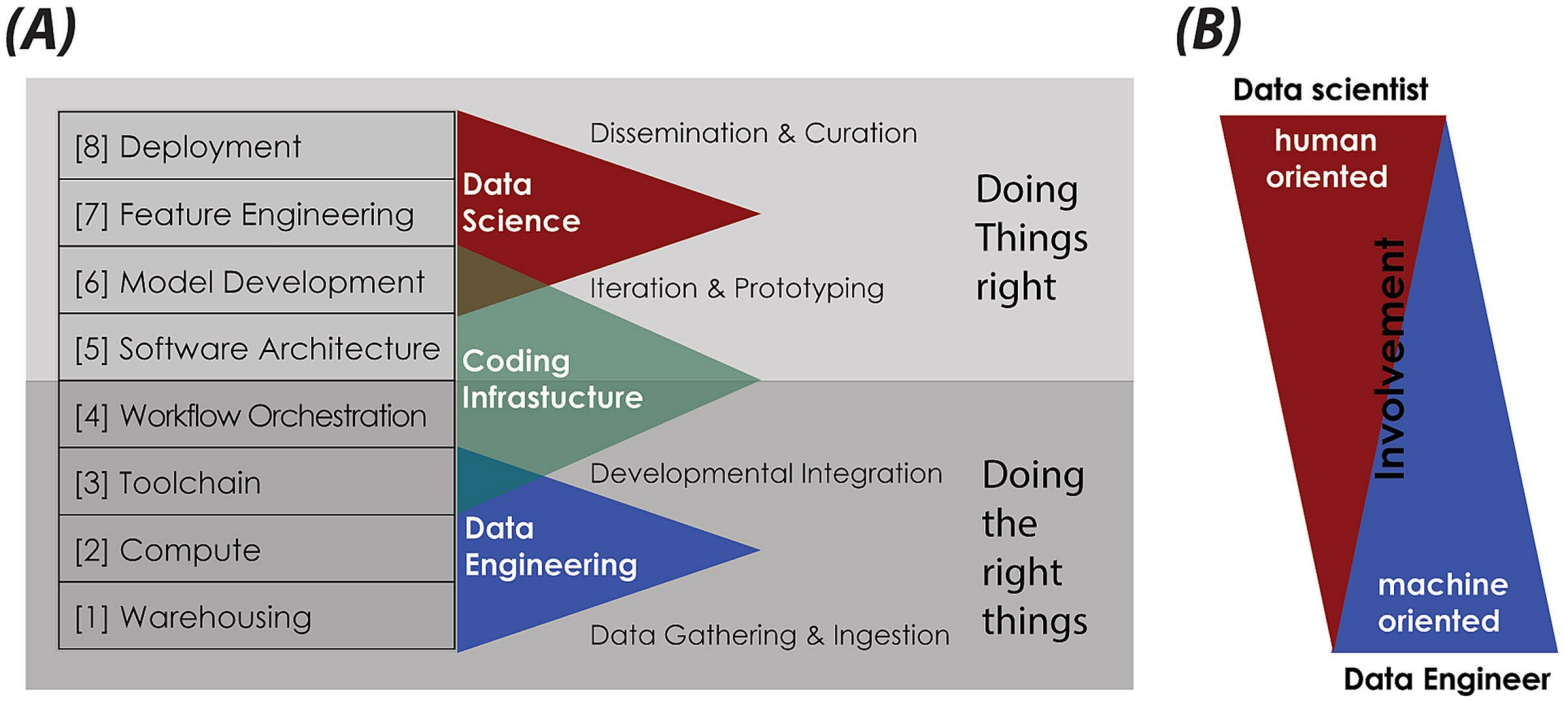
Data science infrastructure (DSI) stack. **(A)** Schematic of the DSI stack, structured as modular, interoperable layers founded on key IT principles: abstraction and modularization, separation of concerns, interoperability and standardization, scalability, and resilience. Each layer—[1] … [8]—fulfils a distinct function, from data storage and processing to analytics and deployment. It supports flexible, maintainable, and scalable data science pipelines. The DSI stack aligns with the Double Diamond design model (https://www.designcouncil.org.uk/our-resources/the-double-diamond/). Lower layers focus on “Doing the right things”—data gathering and integration—, while upper layers emphasize “Doing things right”—curation, iteration, deployment. **(B)** Visualization of the roles and involvement of data scientists versus data engineers across the DSI stack. While data scientists are predominantly active in the human-oriented, upper layers of feature engineering, model development, and deployment, data engineers are primarily engaged in the machine-oriented, foundational layers involving warehousing, compute, and toolchains. This panel highlights the complementary skill sets necessary for an effective and robust data science infrastructure.

At the heart of our methodology is a modular data science infrastructure (DSI) Stack (Figure 1A), designed to organize and streamline the process of generating clinical data by breaking it down into a series of well-defined, interconnected workflows. It starts with ingesting and converting anonymized clinical notes from PDF to Markdown (FLOW01) (see text footnote 4), followed by pseudonymization to protect patient privacy (FLOW02) (see text footnote 4). Synthetic notes are then produced using LLMs, comparing standard prompting (FLOW03) (see text footnote 4) with a Generative Agent approach (FLOW03_AGENT_BASED). The pipeline ends with benchmarking (FLOW04) (see text footnote 4) using metrics for diversity, vocabulary similarity, semantic alignment, and classifier-based machine discernibility—enabling the comparison of the informational and linguistic features of synthetic, anonymized, and real EHRs at both the document and corpus levels. Included is a hands-on guide for responsible deployment of GA-assisted SHDG-workflows,^5^ implemented through open-source data science platforms—Hugging Face Spaces and Flowise—and powered by LLMs accessed via public cloud services such as Azure. This hybrid approach enables rapid prototyping and controlled sharing of workflows, while API key–secured inference endpoints ensure privacy compliance. The GitHub repository also provides lines of example source code (FLOW03) (see text footnote 4) that enables implementation of a privacy-first alternative to public cloud AI services called Ollama, allowing users to run and manage large language models locally while maintaining full data control and privacy compliance.

### 3.1 Data science infrastructure stack

Synthesizing and validating EHRs requires a well-defined data science infrastructure. This involves designing a robust data pipeline, a systematic sequence of processes that transforms raw data into high-quality synthetic datasets and generates actionable insights (Meng, 2021; Tuulos, 2022).

Building a pipeline requires the seamless integration of diverse hardware and software components within a pre-defined DSI stack. In this architecture, each layer—from data ingestion to deployment—builds upon the previous one, creating a cohesive framework. This layered approach streamlines the transformation of real-world EHR samples and clinical practice guidelines into novel, synthesized datasets. By following this structured process, patient privacy and regulatory compliance are maintained, enabling the safe and effective use of synthetic data for research, analytics, and clinical decision support (Priebe et al., 2021; Tuulos, 2022; Hechler et al., 2023).

Our DSI stack—as depicted in Figure 1—is organized into eight layers: [1] Data Warehousing; [2] Compute Resources; [3] Toolchain; [4] Workflow Orchestration; [5] Software Architecture; [6] Model Development; [7] Feature Engineering; [8] Data Product Deployment. The dependency on IT-hardware progressively increases towards the bottom of the stack (Krishnakumar et al., 2023). Next, we describe each of the relevant layers from the bottom up, highlighting their importance and practical application. Note that layers [6] Model Development and [7] Feature Engineering were not applicable to our specific use case and will therefore not be discussed further. Moreover, our DSI stack aligns with the double diamond design model (Kochanowska et al., 2022): lower layers focus on *“Doing the right things,”* while upper layers emphasize *“Doing things right.”*

#### 3.1.1 Pseudonymization

Integrating real-world sample data into the synthesization process enhances the diversity of synthetic data, thereby improving its linguistic quality and informational characteristics to better reflect real-world EHR narratives (Chung et al., 2023). Clinical narratives were collected from 13 patients undergoing treatment for low back pain at a single physiotherapy clinic within a primary care setting, spanning the duration of their therapeutic trajectories. All patients provided informed consent for the use of their EHRs to assist in the generation of synthetic data. All EHRs (N = 13) were manually anonymized by deleting patient names, addresses, social security numbers, contact details, and insurance details.

Pseudonymization served as an essential data pre-processing step prior to warehousing (Section 3.1.2). This involved replacing the names of referring physicians and treating physiotherapists with fictive names, thereby restoring the natural structure of the EHRs. This procedure ensured that sample datasets destined for warehousing were thoroughly de-identified in accordance with Dutch and broader European privacy and regulatory standards. To achieve this, we developed a GenAI-based Named Entity Recognition (NER) workflow, customized for privacy categories, to systematically identify and replace personal identifiers in Markdown files derived from EHR sample PDF documents. Data entry fields for entities such as names, addresses, contact details, birth dates, “burgerservicenummers” (BSNs), insurance details, and financial data were detected and either removed or pseudonymized in compliance with privacy guidelines.

A Jupyter notebook executed custom Python code [GitHub Repository (see text footnote 4): FLOW01] to configure the Azure OpenAI API SDK; submit each document to Azure OpenAI’s GPT-4.1; and apply a tailored system prompt to both identify and pseudonymize specified entities while preserving the Markdown format. Processed files were stored with their original formatting intact for subsequent analysis.

#### 3.1.2 Warehousing

Data warehousing serves as the foundational layer of the DSI stack, providing centralized aggregation and accessibility for static, unstructured datasets. It is crucial for storing both the generated synthetic data (output for developing and testing solutions) and the real-world sample data and clinical practice guidelines (as input knowledge bases for the synthesis process). Besides, the integration of anonymized real-world data from EHR systems, the inclusion of codebooks for labels and abbreviations, and clinical practice guidelines into data warehouses limits chance of hallucination but enhances the diversity, linguistic quality, and therefore the clinical relevance of synthetic data (Chung et al., 2023; Li et al., 2023).

Storing data in accessible formats such as markdown (MD), structured query language (SQL), comma-separated values (CSV), portable document format (PDF), JavaScript object notation (JSON), or images is integral to facilitating interoperability and data sharing within clinical environments. These widely used formats support the storage and exchange of both structured and unstructured data, making it easier for diverse health information systems to work together (Hart et al., 2016). For example, JSON is used in AI-workflows because it is both human-readable and machine-readable. It is particularly helpful in health care and other fields for quickly and efficiently transferring data between systems, applications, or devices.

#### 3.1.3 Compute

The next DSI stack layer is compute, which refers to scalable data processing capacity or computational power. Its purpose is to manage and scale the performance of a predefined set of computational instructions—referred to as a computational workload or task. The type of data science use case dictates its compute requirements, including the need for CPUs, GPUs, TPUs, or internal memory.

To assist the targeted end-users—non-AI specialists—, we decided to employ a hybrid compute solution, combining standard desktop computers or laptops (local computing) with powerful external computing resources available over the internet (public cloud services like Azure, AWS, or Google Cloud). The latter is essential for utilizing state-of-the-art LLMs. These models require specialized high-performance computing hardware and massive computational resources that far exceed the capabilities of standard desktops or laptops.

Public cloud enables scalable LLM deployment with on-demand access to high-end GPUs/TPUs, large memory, and parallel computing, surpassing local infrastructure limits. Since state-of-the-art LLMs are only accessible via cloud-based APIs (Table 1), cloud adoption is essential for high-end performance. However, organizations with sufficient local computing power (e.g., EHR vendors, hospitals) may opt for on-premises GenAI model deployment.

**Table 1:** Key evaluation criteria for selecting LLMs.

Consideration	Explanation	Importance for model selection
Model architecture	The underlying design of the model (e.g., transformer-based, LSTM, etc.).	Influences model capabilities, efficiency, and suitability for specific NLP tasks.
Model size & parameters	Number of parameters indicating model complexity and capacity	Larger models often perform better but require more computational resources; balance needed based on use case.
Inference speed & latency	Time taken to generate outputs during use.	Critical for real-time applications and user experience; faster models enable scalable deployment.
Performance metric scores	Quantitative measures like accuracy, perplexity, BLEU, ROUGE on relevant benchmarks.	Helps objectively compare models’ language understanding and generation quality.
Context window size	Maximum input length (tokens) the model can process at once.	Larger context windows allow handling longer documents or conversations without losing coherence.
Fine-tuning & customizability	Ability to adapt the model to specific domains or tasks via additional training.	Enables tailoring model behavior to unique organizational needs and improves task-specific performance.
Pretraining data & knowledge cutoff	The scope and recency of data the model was trained on.	Determines how current and relevant the model’s knowledge is.
Multimodal capabilities	Support for inputs beyond text, such as images or video.	Expands potential applications, enabling richer interactions and cross-modal understanding.
Use case alignment	Suitability of the model’s strengths to the specific application or domain.	Ensures optimal performance and ROI by matching model capabilities with business goals.
Safety, bias & ethical considerations	Mechanisms to reduce harmful, biased, or inappropriate outputs.	Ensures responsible AI use, compliance with regulations, and trustworthiness.
Licensing & accessibility	Terms of use, availability (open source vs. proprietary), and cost implications.	Affects budget, deployment flexibility, and compliance with organizational policies.
Ecosystem & integration	Availability of APIs, developer tools, and compatibility with existing systems.	Facilitates easier implementation, faster development cycles, and operational efficiency.
Enterprise readiness	Support for scalability, data privacy, user data control, and cloud provider support.	Important for secure, compliant, and robust deployment in production environments.

Listed are essential considerations—including usability, ethical implications, and enterprise requirements—compiled together to assist non-AI specialists in selecting an LLM that aligns with their specific needs. The selected criteria are based on a synthesis of recent expert analyses and practitioner frameworks.

#### 3.1.4 Toolchain

The toolchain layer ensures the correct functioning of the desired workflow orchestration. Our protocol leverages rapid prototyping platforms to synthesize and validate EHRs using state-of-the-art GA-assisted SHDG workflows. These platforms offer an intuitive drag-and-drop user interface, simplifying implementation by eliminating the need for data engineering expertise in LLM deployment. This makes GenAI-technology more accessible for non-AI specialists by facilitating browser-based access (Kumar, 2023; Jeong, 2025).

We implemented a Docker-based^6^ architecture to enable secure, reproducible workflows across on-premises and public cloud environments. By deploying containerized workflows via API key–secured inference endpoints, we ensure scalable resource allocation, consistent performance, and robust access control (Abhishek and Rao, 2021; Ait et al., 2025). Inference endpoints offer a user-friendly interface for submitting inputs—such as prompts for synthetic EHR generation—and receiving outputs, allowing on-demand use of custom GA-assisted workflows without exposing users to underlying infrastructure or model complexity (Fu et al., 2025; Gupta, 2025).

Hugging Face Spaces^7^ offers a public cloud platform for rapidly building, sharing, and interacting with containerized GenAI applications through intuitive interfaces, automatically hosting workflows at a public URL. This supports Docker-based deployment of low-code/no-code tools such as Flowise, Langflow and AutoGen for developing multi-agent AI workflows (Kumar, 2023; Jeong, 2025). Flowise enables users to visually compose, configure, and deploy LLMs without programming expertise, while Langflow allows for direct code customization. AutoGen Studio facilitates rapid prototyping and orchestration of LLM-based multi-agent systems via a low-code Python framework. For example, an implementation guide titled “Learn how to deploy Flowise on Hugging Face” is available online.^8^

The usefulness of Open-source LLMs like LLaMA, Qwen, DeepSeek, and Phi in clinical settings is hampered by the frequent production of unsupported facts, contradictions, and omissions—collectively known as hallucinations—which present a substantial safety risk. Evaluation of the MIMIC-IV dataset revealed that while these models can accurately capture up to 83% of admission reasons and key events, their performance dropped dramatically for critical follow-up recommendations, with comprehensive coverage as low as 29% (Das et al., 2025).

Considering both the above outlined limitations and the selection criteria presented in Table 1, we selected OpenAI’s GPT-4.1 (version:2025-04-14) as our preferred LLM. GPT-4.1 offers a one million token context window, which allows for comprehensive analysis of extensive patient records, and demonstrates robust instruction-following and advanced reasoning abilities (OpenAI, 2025). The criteria listed in Table 1 reflect insights drawn from recent expert reviews and leading practitioner models (QuantSpark, 2023; Inoue, 2024; Ruczynski, 2024; Chojnacki, 2025; Morris et al., 2025).

In addition, GPT-4.1 incorporates advanced domain adaptation, sophisticated fact-checking mechanisms, and alignment strategies to reduce hallucination rates and enhance faithful adherence to source texts. Here, *“Advanced domain adaptation”* refers to the ability of an LLM to adjust its understanding and generation of text to fit the specific language, conventions, and knowledge of a particular field or domain—such as medicine (Singhal et al., 2023). Moreover, GPT-4.1 appears to demonstrate advanced comprehension of medical and healthcare language, enabling more accurate interpretation of complex EHR narratives and improved detection of inconsistencies (Walturn, 2025).

#### 3.1.5 Workflow orchestration

**Figure 2:**
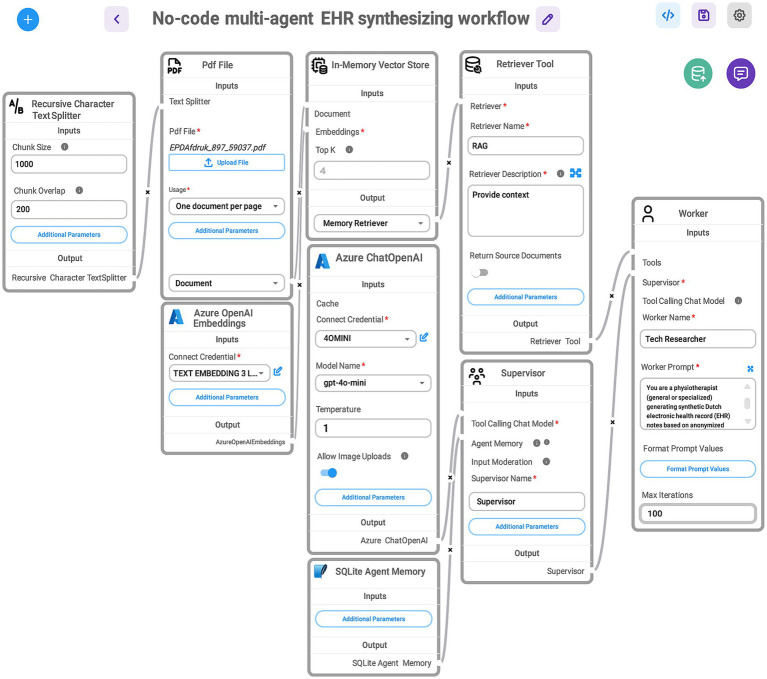
Visual representation of a no-code, multi-agent workflow for synthesizing EHRs. Data flows through connected tools and agents, enabling an iterative, structured generation process without manual coding. The here shown GA-assisted SHDG workflow begins with a Recursive Character Text Splitter that divides the uploaded PDF file containing anonymized EHR data into manageable chunks. These chunks are processed using Azure OpenAI Embeddings and stored in an In-Memory Vector Store. A Retriever Tool (RAG) then queries the stored embeddings to provide relevant context. The Azure ChatOpenAI component, configured with the GPT-4.0-mini model, interacts with stored agent memory (SQLite Agent Memory) and coordinates with two agents: the Supervisor—acting as a senior physiotherapist specialized in low back pain—who manages task instructions and workflow control, and directs the Tech Researcher—acting as a practicing physiotherapist (general or specialized—who executes prompts to generate synthetic Dutch-language EHR notes). Note: The workflow—accessed via a web interface—maintains contextual memory across user queries for seamless, multi-turn interactions and stops automatically when the supervisor determines completion. This design enables rapid prototyping of SHDG solutions by healthcare researchers and practitioners without requiring advanced expertise in AI. For more information on the adopted technologies and their implementation, see the Toolchain Section (Section 3.1.4 of the DSI stack).

To synthesize Dutch clinical narratives related to low back pain in physiotherapy using genuine EHRs, we developed a GA-assisted, no-code workflow built on a rapid prototyping platform that allows end users to quickly construct and test GenAI solutions (Figure 2). This platform is organized into modular components—known as modules—each serving a specific purpose and designed to be easily rearranged or modified.

**Figure 3:**
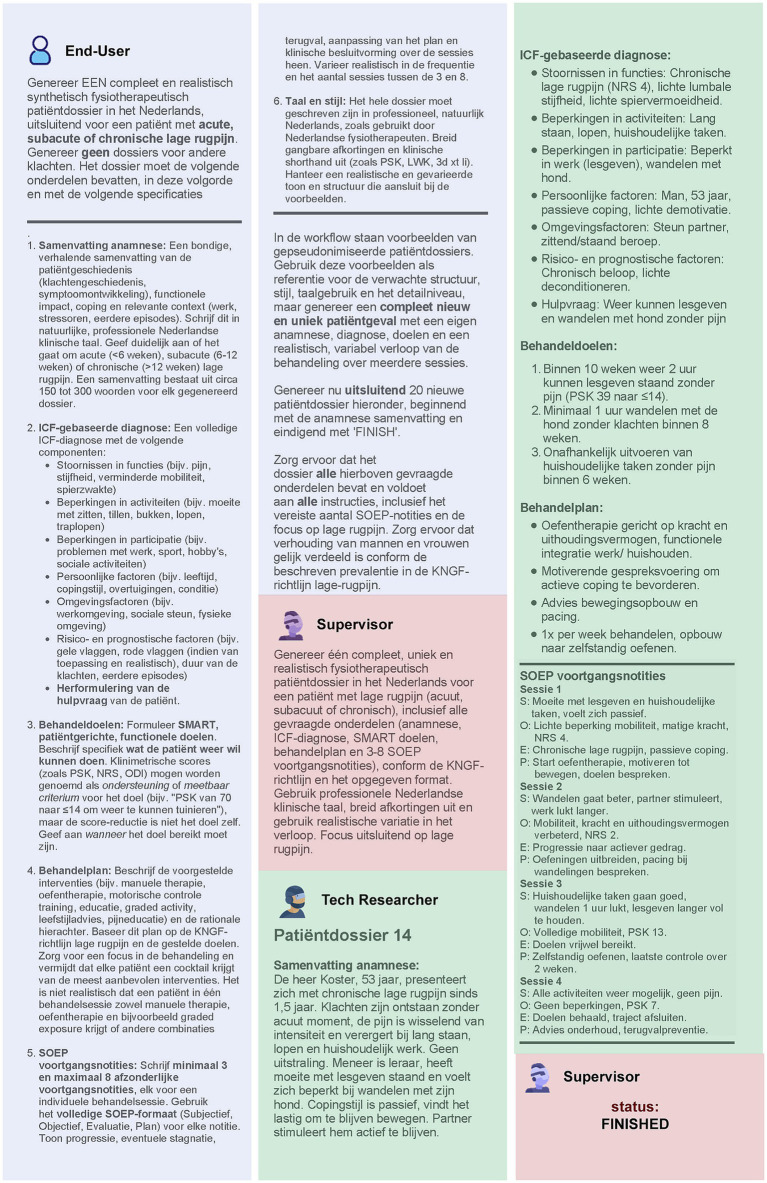
Example of a single-turn input/output interaction when applying the multi-agent workflow for generating realistic, structured EHRs, as described in Figure 2. In this scenario, the End User—a practicing physiotherapist—uses a web-based interface to request the creation of 20 synthetic but realistic EHRs in Dutch. The request specifies detailed content and formatting requirements, including: a concise patient history summary, a clearly stated help-seeking question, an ICF-based diagnosis, measurable treatment goals, and a treatment plan aligned with KNGF guidelines. All records must use professional Dutch clinical language with correct abbreviations. The Supervisor ensures these specifications are complete and unambiguous before the Tech Researcher produces a synthetic yet realistic Dutch-language EHR. Each record contains the requested summary, patient demographics, presenting complaint, ICF-based functional and contextual factors, SMART goals, an individualized treatment plan, and SOAP-formatted progress notes. For illustration purposes, only Patient Dossier 14 from the generated set is shown here. The Supervisor reviews this output, confirms it meets all requirements, and marks the task as finished. *Color coding:* Blue—End User; Pink—Supervisor Agent; Green—Tech Researcher (worker Agent).

Central to our approach is a multi-agent architecture (Figure 3), comprising a supervisor agent and one or more worker agents, each guided by tailored prompts that correspond to their unique responsibilities. To make the process intuitive, we utilized a “What-IF” scenario: imagine a clinical team with a general practitioner, a specialist, and a medical scribe collaborating to create a realistic synthetic patient note. In this analogy, the supervisor agent acts like the lead clinician—overseeing the entire process, distributing tasks, prioritizing activities, monitoring progress, and ensuring the workflow stays on track and determines when the task is finished—while the worker agents take on specialized roles such as drafting clinical content or formatting records, analogous to the scribe and specialist. Each agent receives role-specific guidance through prompt engineering (Chen et al., 2025a), ensuring adherence to clinical standards and the accurate, coherent assembly of narratives. This modular, role-based structure not only enables seamless coordination and iterative refinement among AI agents, but also produces synthetic EHRs with high clinical validity and practical value for healthcare workers.

##### 3.1.5.1 Supervisor agent prompt

The supervisor agent was equipped with a *“system prompt”*—a foundational set of instructions that defines the LLM’s overarching persona, scope, and governance strategy. Specifically, this prompt directed the supervisor agent to represent an experienced physiotherapist overseeing the clinical perspective of a registered physiotherapist tasked with generating authentic Dutch EHRs for low back pain cases. The prompt specified how to generate clinical documentation according to the International Classification of Functioning, Disability and Health (ICF) framework domains (World Health Organization, 2001), and adherence to the Dutch Royal Society for Physiotherapy (KNGF) guideline on low back pain (Swart et al., 2021). This ensured that every generated record aligned with current best practices in physiotherapy documentation.

The prompt further instructed the supervisor agent to restrict outputs solely to the requested EHR content, thereby enforcing compliance and preventing the inclusion of extraneous or sensitive information. Functionally, it mandated the supervisor agent to orchestrate the division of labor among worker agents, manage task handoff, establish execution priorities, consolidate contributions, and verify completion of all record elements in accordance with professional documentation standards.

##### 3.1.5.2 Worker agent prompt

Under the coordination of the supervisor agent, each worker agent received individualized “worker prompts” tailored to its specialized domain within the workflow. These prompts offered detailed, task-specific instructions for generating a single EHR taking into account the clinical nuances of physiotherapy care for (sub)acute or chronic low back pain, as well as relevant and documentation standards (Driehuis et al., 2019; Swart et al., 2021). Most notably, the worker prompts required structured output, as listed in Table 2.

**Table 2:** Targeted worker agent prompting.

Consideration	Explanation
Anamnesis summary	Craft a concise, professional account of the patient’s medical history, the impact of symptoms, coping mechanisms, and clinical context; ensure precise specification of symptom duration (acute, subacute, or chronic) and maintain professional standards of written Dutch.
Physical therapy diagnosis	Deliver a comprehensive, multidimensional diagnostic formulation, detailing impairments, activity limitations, participation restrictions, relevant contextual (personal and environmental) factors, risk/prognostic indicators, and a reformulation of the patient’s explicit help seeking question—all mapped to ICF domains.
Treatment goals	Articulate SMART (Specific, Measurable, Achievable, Relevant, Time-bound), patient-centered, and function-oriented short- and long-term goals, with reference to clinical metrics (e.g., NPRS, QLBDS) strictly as criteria, not as goals themselves. Target dates for each goal are specified according to best practice.
Treatment plan	Compose an intervention strategy, incorporating manual therapy, exercise programs, educational components, psychosomatic physiotherapy and other modalities, substantiated by the KNGF guidelines and explicitly related to the established treatment goals.
SOEP progress notes	Generate between three and eight detailed progress notes, each corresponding to an individual treatment session, structured in the SOEP (Subjective, Objective, Evaluation, Plan) format. These notes are intended to reflect realistic clinical variation, including both therapeutic progression and stagnation or need for adjustment.
Language and style	All documentation must be rendered in idiomatic, professional Dutch with expanded abbreviations (e.g., MT, NPRS, LBP), and maintain a narrative and tone that emulates authentic Dutch physiotherapy records as demonstrated in the reference examples.
Referencing examples and output format	Worker Agents are instructed to use pseudonymized sample EHRs solely as a stylistic and structural reference, ensuring every generated dossier remains unique.

Detailed are the specific domains addressed by the worker agent, along with corresponding explanations, to guide the synthesis of clinically valid and contextually appropriate physiotherapy records for low back pain.

#### 3.1.6 Software architecture and deployment

Data product deployment is the final layer of our DSI stack, where prototype workflows transform into web-accessible applications. This layer ensures that data products—such as LLM GPT-4.1 and Hugging Face Spaces—are securely and reliably made available through Inference Endpoints secured with API key authorization (as was discussed in the Toolchain Section 3.1.4). This also protects patient privacy and complies with data management and regulatory standards, including the GDPR^9^ and the EU AI Act^10^ (Haug, 2018; Hoofnagle et al., 2019; European Parliament and Council, 2024). Additionally, it manages resources and access controls to maintain data security and organization. By simplifying deployment through no-code/low-code platforms, discussed in the next section, with reusable components and clear specifications, this layer helps deliver trustworthy, easy-to-use solutions that support clinical decision-making, research, and analytics.

### 3.2 No-code proof-of-concept

This section details a rapid-prototyping implementation to demonstrate the feasibility and effectiveness of generating synthetic EHRs using GA-assisted SHDG workflows (Figures 2, 3). By combining modularity and no-code principles, our proof-of-concept illustrates a practical, user-friendly approach for healthcare professionals to generate, validate, and experiment with synthetic health data. It supports iterative development, transparency, and ease of integration with other systems or data science workflows.

Here, we outline the specific tools and configurations used, illustrating how the DSI stack layers translate a specific data science use case into a functional workflow. The protocol presented here serves as a practical step-by-step guide for replicating our approach and showcases the capabilities of the proposed architecture in addressing the challenges of clinical text synthesis.

For demonstrative purposes, we utilized public Hugging Face Spaces infrastructure in combination with Flowise to facilitate deployment (see Section 3.1.4). This setup allows custom-made workflows to be shared publicly or privately, making them accessible via a web interface or API, and supports secure credential management for connecting to external LLMs and API services.

The workflow (Figure 2) to synthesize EHRs, required additional modules for document ingestion, embedding generation (using Azure API key credentials), agent memory management (using a SQLite database) and memory retrieval through a vector store. Table 3 provides a functional overview of the Flowise modules shown in Figure 3. To digest unstructured sample data (e.g., PDFs), we applied a recursive character text splitter. Traditionally, parameters such as chunk size and chunk overlap are critical: smaller chunks (500–1,000 characters) improve granularity but may fragment context, while larger chunks (>1,500 characters) support coherence but risk exceeding model context windows. Moderate overlap (100–200 characters) helps maintain semantic continuity between segments. However, recent studies suggest that late chunking—where segmentation occurs after the model embedding step rather than before—can preserve global context more effectively and enhance downstream performance, particularly in retrieval-augmented tasks (Günther et al., 2024).

**Table 3:** Workflow stages, modules, and operational details for no-code GA-assisted synthetic EHR processing.

Workflow stage	Module	Operational details
Ingestion and preprocessing parsing	A/B recursive character text splitter	**Function:** Splits a large text document into semantically meaningful “chunks” (e.g., 1,000 characters / 200-character overlap) to meet processing constraints. **Input:** Large text document (e.g., PDF file). **Output:** Prepared data chunks for downstream processing within input size limits.
	PDF File	**Function:** Extracts text content from uploaded PDFs. **Input:** Text splitter output; end-user uploads a PDF file (e.g., a real-world EHR). **Output:** Structured text format suitable for processing (one document per page).
	In-memoryvector store	**Function:** Stores embeddings for fast semantic EHR searching and referencing in agent workflows. **Input:** Embeddings (via Azure OpenAI) derived from document chunks (e.g., from PDF files). **Output:** Embeddings stored for quick retrieval.
	Retriever tool	**Function:** Queries the vector store to retrieve relevant EHR content based on prompts or keywords. **Input:** Query from workflow (e.g., “provide context”). **Output:** Most contextually relevant EHR chunks for the next workflow step.
Embeddings	Azure OpenAI embeddings	**Function:** Generates numerical vector representations of text chunks for similarity search and LLM processing. **Input:** Name and credentials for the text embedder (e.g., text-embedding-3-large). **Output:** Numerical embeddings of text chunks.
Agent memory management	SQLite agent memory	**Function:** Maintains multi-turn interaction memory for context continuity. **Input:** Additional parameters (if any). **Output:** Persistent memory of conversation history, prior actions, and current state.
Agent orchestration of multi-turn interactions	Supervisor	**Function:** Central controller — interprets tasks, routes them to workers or tools, ensures proper sequencing, and coordinates memory and moderation. **Input:** LLM (e.g., GPT-4o-mini), agent memory, supervisor prompt/role (plus optional parameters). **Output:** Coordinated orchestration between tools, agents, and workflow steps.
	Worker	**Function:** Executes reasoning, analysis, or synthesis tasks using retrieved context and domain knowledge. **Input:** Tools (via supervisor), worker prompt/role (plus optional parameters). **Output:** Structured answers, summaries, or insights per prompt.
User interaction and language model reasoning	Azure ChatOpenAI	**Function:** Provides a conversational interface between user and workflow, leveraging memory and context for responses. **Input:** Prompts from the user with parameters (e.g., credentials, temperature, mode name). **Output:** User-facing responses and orchestrated agent actions.

We employed GPT-4.1 LLMs—using Azure API key credentials—for supervised reasoning and text generation. A key model parameter for any LLM is temperature, which controls generative diversity: lower values (~0.2–0.4) yield deterministic, guideline-conform output, while higher values (~0.7–0.9) promote creative variability (Peeperkorn et al., 2024). For clinical synthesis, we assumed that a temperature between 0.3–0.5 best balances realism and consistency; however, future experiments are required to empirically evaluate model performance at varying settings. The *“agentflow”* as implemented within the Flowise no-code framework can be downloaded as a JSON file from our GitHub Repository (see text footnote 1) (GA-assisted SHDG workflow).

The supervisor agent represents an experienced physiotherapist who ensures that documentation conforms to ICF domains, linguistic plausibility, and overall fidelity (Figure 3). The worker agent (Figure 3) embodies one of several physiotherapy profiles (e.g., generalist, manual therapist, exercise therapist, psychosomatic physiotherapist) and is tasked with generating synthetic clinical narratives. To reduce the risk of hallucination and ensure domain-conformant output, we implemented a retrieval-augmented generation (RAG) pipeline. This supported the contextual grounding of worker agent output using a vectorized memory store filled with real-world sample data, clinical practice guidelines, and documentation standards used by Dutch physiotherapists. This architecture allows agents to generate clinically realistic output grounded in both empirical input and normative context (Chung et al., 2023; Li et al., 2023).

### 3.3 SHDG automation through GenAI-assisted co-development

Here we describe how we automatized the entire synthetic EHR pipeline; using a novel engineering approach called: GenAI-assisted co-development. This technique fosters collaboration between human developers and GAs (Park et al., 2023) allowing human domain specialists to iteratively refine code through natural language prompts and AI-tools like GitHub Copilot, Perplexity, and Gemini. Copilot helps with coding by suggesting and writing code, Perplexity helps find answers by providing clear, sourced information, and Gemini acts as an all-around smart assistant that can respond to questions, summarize text, and assist with a variety of tasks. As such, GenAI-assisted co-development fundamentally redefines the paradigm of human-AI collaboration (Orru et al., 2023; Ulfsnes et al., 2024; Casper et al., 2025; Mayo, 2025).

For instance, GAs like AlphaEvolve (DeepMind, 2025) exemplify the power of GenAI-assisted co-development by autonomously facilitating iterative code improvement. AlphaEvolve operates as an evolutionary coding agent that leverages the orchestration of multiple LLMs, enabling ongoing refinement of algorithmic solutions through a cycle of edits and evaluator feedback. This process mirrors the collaborative workflow described earlier (Section 3.2), whereby human expertise and GAs interleave in a continuous dialogue—using natural language guidance to steer, critique, and optimize computational problem-solving. In this way, AlphaEvolve and similar systems advance the core objective of our methodology: to solve complex scientific and computational challenges by seamlessly integrating human insight with autonomous generative capabilities (Novikov et al., 2025).

#### 3.3.1 GA-assisted SHDG workflow validation

We started by confirming that our GA-assisted SHDG workflow (Section 3.2) was effective in generating meaningful synthetic EHRs. This preliminary validation step involved human specialists (authors MV, MS) assessing the synthetic EHR samples for realism, internal coherence, and adherence to professional clinical documentation standards. We confirmed that the generated records were representative for deployment in downstream healthcare applications and research.

#### 3.3.2 PDF-to-Markdown conversion

As a foundational step, we used Gemini 2.5 Flash as the core LLM in our GenAI-Assisted Software Development workflow to generate and refine Python code—contained in Jupyter notebooks—for automating PDF-to-Markdown conversion. Guided by natural language prompts written by human domain experts, Gemini 2.5 Flash authored code that extracts text from PDFs (via Python packages such as OpenAI, PyMuPDF, and glob), interfaces with Azure OpenAI’s GPT-4.1 for Markdown formatting, and supports batch processing of files. This collaborative approach enabled efficient, maintainable code development, combining Gemini 2.5 Flash’s reasoning and coding abilities with GPT-4.1’s language understanding to deliver scalable and accurate document conversion. Note, Human supervision— following a human-in-the-loop approach, in which humans remain actively involved in reviewing, verifying, and refining AI outputs—was essential to ensure that the AI-generated code functioned correctly. The code used is available online via our GitHub Repository (see text footnote 1) (FLOW01).

#### 3.3.3 Pseudonymization

We prompted Gemini 2.5 Flash to integrate a pseudonymization step using a second call. This involved a second call to the Azure OpenAI GPT-4.1 API with a tailored system prompt designed to identify and pseudonymize specified named entities while preserving the Markdown format. For details about the selected named entities, we refer to Section 3.1.1. The code used is available online via our GitHub Repository (see text footnote 1) (FLOW02).

#### 3.3.4 EHR-synthesis co-developed with GenAI technology

Subsequently, we instructed Gemini 2.5 Flash to generate Python code that implements the synthetic data generation process. This process used the pseudonymized Markdown files as contextual examples for GPT-4.1, guided by the natural language prompts previously specified for the supervisor agent and worker agents (see Section 3.1.5). A comprehensive explanation of the code is provided, and it is publicly available as a downloadable Jupyter notebook from our GitHub Repository (see text footnote 1) (FLOW03).

#### 3.3.5 Benchmark framework & analysis

Finally, Gemini 2.5 Flash was tasked with developing a programmatic approach to assess how closely synthetic data mirrors the informational and linguistic characteristics of pseudonymized real-world EHRs. This assessment strictly adheres to an evaluation framework comprising ten distinct metrics, as detailed in the next Section 3.4, to evaluate the indistinguishability of synthetic from real data. The code used is available online via our GitHub Repository (see text footnote 1) (FLOW04).

### 3.4 Quantitative assessment of synthetic data quality

To systematically assess the fidelity of GA-assisted SHDG, we recognized that no single metric would suffice. Therefore, we selected ten distinct metrics to jointly evaluate both the informational and linguistic qualities of clinical documents on both the individual document and corpus levels. These metrics, summarized in Tables 4, 5, were chosen to provide clear and quantifiable assessments that are informative for both AI specialists and clinical experts.

**Table 4:** Metrics used to evaluate surface-level similarities.

Metric	Category	Purpose	Interpretation (desired score)	Interpretation (undesired score)
Average word count	Structural fidelity	Compares word count per document between datasets.	Similar average word counts between synthetic and real data.	Consistent divergence (higher/lower) in average word count, suggesting content over/under-generation.
Average unique word count	Linguistic	Compares the diversity of vocabulary per document.	Similar average unique word counts, indicating comparable linguistic richness.	Significant differences, suggesting issues with vocabulary diversity.
Average document length (characters)	Structural fidelity	Compares overall document size in characters.	Similar average document lengths between synthetic and real data.	Consistent divergence (higher/lower) in average document length, suggesting content over/under-generation.

Structural and linguistic metrics for evaluating similarities between original, pseudonymized, and synthetic documents, with interpretation guidelines for desirable and undesirable results.

**Table 5:** Metrics used to evaluate linguistic and information level similarities.

Metric	Category	Purpose	Interpretation (desired score)	Interpretation (undesired score)
Shannon’s entropy (characters)	Textual diversity	Quantifies richness and unpredictability at character level for the entire corpus.	Similar entropy values to real data.	Much lower entropy (overly repetitive) or much higher entropy (excessively random/incoherent).
Shannon’s entropy (words)	Textual diversity	Quantifies richness and unpredictability at word level for the entire corpus.	Similar entropy values to real data, indicating comparable vocabulary diversity.	Much lower entropy (overly repetitive vocabulary) or much higher entropy (excessively random/incoherent word choice).
Jensen-Shannon divergence	Word distribution similarity	Measures the statistical distance between word probability distributions of two corpora (range 0–1).	Low JSD (closer to 0), implying similar word frequency patterns and vocabulary overlap.	High JSD (closer to 1), indicating marked differences in vocabulary or word usage patterns.
Average Bigram Pointwise Mutual Information(PMI)	Naturalness of word associations	Quantifies the average strength of association between adjacent words.	Comparable PMI, indicates synthetic text mimics natural bigram.	Significant differences, suggesting unnatural word pairings or phrasings.
BLEU score	Lexical similarity / surface-level overlap	Quantifies n-gram overlap between synthetic and reference texts (range 0–100).	Higher BLEU score (closer to 100), indicating greater literal overlap in n-grams.	Lower BLEU score (e.g., 4.6), indicating very low literal overlap; suggests synthetic text does not closely replicate exact phrasing, potentially acceptable if novelty is a goal.
BERTScore	Semantic alignment	Assesses semantic similarity using contextual embeddings (F1 typically 0–1).	High F1 score (closer to 1), indicating strong semantic alignment and meaning preservation.	Lower scores suggest synthetic data differs from the sample data. This indicates the newly generated data is not an exact replica of its origin
Classifier performance (AUC)/(AUPRC)	Inseparability	Tests how easily a classifier can distinguish real (pseudonymized) from synthetic data. Indicates the “realism” of synthetic data. (range 0–1).	AUC/AUPRC approaches 0.5, implying classifier cannot effectively differentiate (high mimicry). Lower values are desirable for synthetic data quality.	AUC/AUPRC ≈ 1.0: Classifier easily separates classes; unrealistic synthetic data. Good indication of “machine-discernibility.” Limitations: Sensitive to dataset size Depends on classifier choice

Metrics assessing overall similarity between real and synthetic clinical text corpora—including diversity, vocabulary, semantic alignment, and machine discernibility—emphasizing that desired scores reflect close corpus-level resemblance across these dimensions.

Our framework goes beyond surface-level resemblance—such as basic structure—by also examining deeper linguistic and semantic properties. Specifically, we evaluate whether synthetic texts authentically mirror genuine EHRs in their phrasing, stylistic features, and conveyed meanings. To ensure this, we analyzed pooled corpora of pseudonymized real and synthetic data (Table 5), assessing similarities in word sequences, style, and intent.

Detailed implementation notes, equations and code for each metric are available in our public GitHub Repository (see text footnote 1) (FLOW04).

#### 3.4.1 Document level assessment

Three different metrics —the original clinical documents, their pseudonymized counterparts, and synthetic documents generated through the GA-assisted SHDG process—were used to provide insight into the structural fidelity of the generated documents, ensuring that basic textual characteristics were faithfully reproduced in the synthetic samples. Assessment of averaged word count, average unique word count, and average document length (measured in characters) provided a straightforward means for comparing the overall size and composition of documents between the original, pseudonymized, and synthetic datasets.

Table 4 provides an overview of structural and linguistic metrics used to evaluate the fidelity of GA-assisted SHDG at the document level. It specifically focuses on comparing the original, pseudonymized, and synthetic documents in terms of word count, vocabulary diversity, and document length. The table offers guidance for interpreting whether the observed scores indicate that synthetic documents adequately replicate the structure and linguistic richness of the real data or reveal potential discrepancies.

Collectively, the document-level metrics of Table 4 allow for direct comparison of volume and informational content across the three datasets. Consistent differences in document length, word count, or vocabulary diversity between synthetic and real documents can signal problems in the data generation process, such as systematic under- or over-generation. Early detection of such discrepancies enables targeted improvements, ensuring that synthetic data more accurately reflects the completeness and verbosity found in authentic datasets.

#### 3.4.2 Corpus level assessment

The inherent heterogeneity in EHRs—stemming from both the variety in patients and the diversity in documentation by healthcare professionals—can greatly impact comparability between real and synthetic data. Furthermore, as the aim of synthetic data is often to generate more data than is originally available (thus overcoming limited data availability), metrics other than pairwise comparison of individual documents are needed to evaluate the linguistic and informational similarities between datasets. To assess the actual comparability between the real pseudonymized and the synthetic clinical text, we pooled the individual documents into two corpora.

Table 5 provides a comprehensive set of metrics for assessing the fidelity of synthetic clinical text at the corpus level. Given the inherent variability in real-world electronic health records and the goal of creating synthetic datasets that closely resemble the originals, these metrics move beyond simple pairwise document comparisons to evaluate broader linguistic and informational features. The table outlines measures of textual diversity, vocabulary similarity, semantic alignment, and machine discernibility, each with its specific interpretation. Collectively, these metrics enable a holistic evaluation of how well the synthetic corpus replicates the complexity, nuance, and realism of the real clinical text, providing an in-depth view of corpus-level similarity across multiple dimensions.

### 3.5 Sample dataset description

The original dataset comprised N = 13 EHRs in PDF format, all relating to Dutch patients suffering from lower back pain. These real-world documents featured a combination of structured and unstructured text, including clinical notes, reports, and other pertinent patient information. To characterize these EHRs in terms of linguistic quality and informational content, a custom Python script was developed through GenAI-assisted co-development (see Section 3.3 for a detailed description).

For each file, various parameters were extracted and calculated, including *storage size* (in MB), *textual content size* (measured as total words, unique words after tokenization and lowercasing, and total characters as a measure of document length), and the primary language detected within the textual content. *Structural elements* such as the presence and count of tables, figures (images), and annotations were also identified. Additionally, *Shannon entropy* was computed at both the character and word level to quantify the average uncertainty or randomness in the text, thereby providing insight into its complexity and predictability. The *canonical Jensen-Shannon Divergence (JSD)* was calculated to compare the word distribution of each document with the overall word distribution across the dataset, reflecting the distinctiveness of each document’s language use. Finally, the *average pointwise mutual information (PMI)* for word bigrams was determined to assess the strength of association between commonly co-occurring words. Note, the use of PMI was inspired by the mutual information approach used in the neurophysiology study by van der Willigen et al. (2024), which measures the dependence between complex spectral-temporal sound representations. Both methods apply principles of information theory to quantify meaningful relationships related to NLP, though in distinct data domains (language vs. auditory processing) and at different scales (word pairs vs. neural coding of sound features).

## 4 Results

We start by reporting on a document-level assessment (Section 4.1), examining whether the generated data are contextually and semantically consistent with real EHRs. This is followed by a corpus-level assessment (Section 4.2) to assess whether our GA-assisted SHDG protocol adhered to established clinical data standards and preserved the statistical properties of the sample dataset (for details, see Section 3.5).

### 4.1 Document level assessment

**Figure 4:**
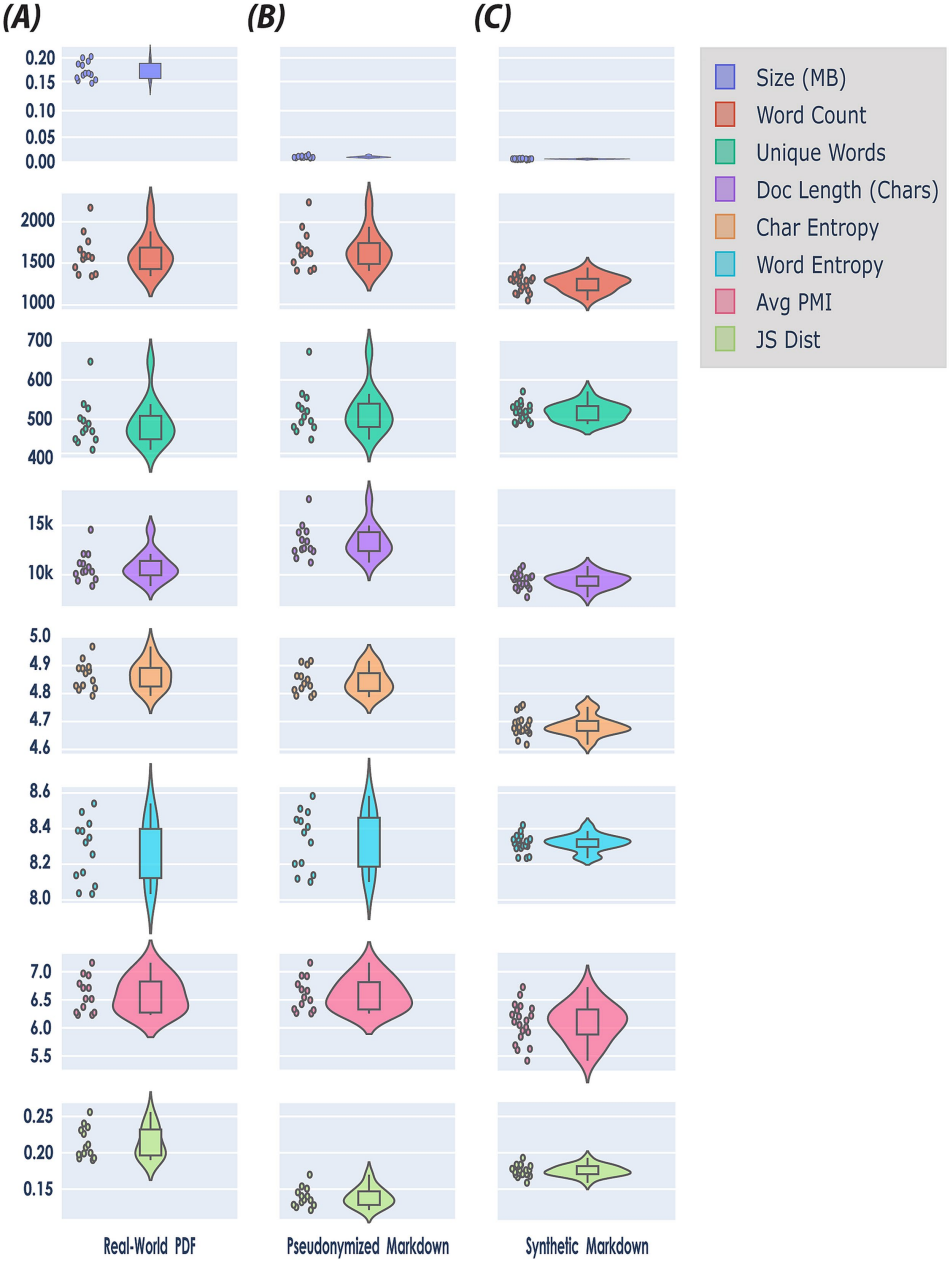
Document level assessment using a holistic benchmark framework for quantitative evaluation of synthetic data quality (see Table 4). The figure presents a matrix of violin plots comparing the distributions of eight features—Size (MB), Word Count, Unique Words, Document Length (Chars), Character Entropy, Word Entropy, Average Pointwise Mutual Information (PMI), and Jensen-Shannon (JS) Distance—across three datasets: **(A)** real-world PDF (*N* = 13), **(B)** pseudonymized markdown (*N* = 13), and **(C)** synthetic markdown (*N* = 20). Note, each feature is encoded by a unique color for visual clarity (see Legend upper right side of the figure). Violin plots combine aspects of box plots and kernel density plots to provide a nuanced visualization of distributional characteristics. Specifically, the width of each violin at a given value represents the estimated probability density of the data at that value, as calculated by a kernel density estimator. This allows for the depiction of multimodality, skewness, and overall distributional shape, beyond summary statistics such as mean or quartiles. In this matrix, each subplot includes both the violin plot and overlaid scatter points indicating individual data instances, thereby facilitating both distributional and sample-level comparison of features across the three datasets.

The first 4 rows of Figure 4—Size (MB), Word Count, Unique Words, Document Length (Chars)—provide a structural element characterization of the original real-world, pseudonymized, and synthetic datasets, respectively. Each real-world EHR document—provided in PDF format—was analyzed for both linguistic quality and informational content. Structural document components—such as tables and images—were also quantified; across the original EHRs, between two and four tables were identified per file, while no figures (images) were detected in any document.

When converting the original, real-world sample data from PDF to pseudonymised Markdown format, file sizes dropped noticeably. This is because PDF files retain a large amount of information—including embedded fonts, images, and detailed layout instructions—to ensure consistent appearance across devices. In contrast, Markdown files contain only the essential textual content and minimal formatting information, omitting media and complex layout data, which makes them much more compact (first row, Figure 4), However, during the pseudonymization phase, we added information to the documents (see Section 3.1.1). Consequently, the word count, and document length of the pseudonymized data increased somewhat (second row, Figure 4), reflecting the inclusion of additional descriptive tokens and labels in the file.

The overall structure of the synthetic data differed substantially from the pseudonymized data: on average, synthetic clinical notes were approximately 30% shorter in length (9,412 vs. 13,370). This reduction suggests that synthetic documents contained significantly less content per record, potentially omitting important clinical details or context. While real-world EHRs often include duplicate or redundant information (Nijor et al., 2022; Steinkamp et al., 2022), we observed that the synthetic data lacked such redundancy. This absence likely accounts for the notable decrease in word count and document length in the synthetic notes, even though the number of unique words remained similar between the two datasets.

At the per-document level, the mean bigram PMI was also slightly lower for synthetic documents (6.26) compared to pseudonymized documents (6.40); however, this difference was not statistically significant (Mann–Whitney U = 161.00, *p* = 0.2611). This suggests that although, on average, synthetic texts have somewhat weaker word pairings, individual documents do not consistently differ in bigram association strength, indicating that variability in bigram usage exists within both pseudonymized and synthetic documents.

### 4.2 Corpus level assessment

We compared our synthetic clinical text to the real-world, pseudonymized clinical notes at corpus level (Section 4.2) using informational and linguistic measures, including Shannon’s Entropy and average bigram PMI. The results are shown in Table 6.

**Table 6:** Corpus level assessment: pseudonymized (Pseudo) versus synthetic (Synth) text corpora.

Metric *interpretation*	Pseudo mean	Synth mean	Mann-whitney U (U-stat)	*p*-value
Textual diversity metrics				
Corpus shannon entropy (character) *Pseudo > Synth character diversity*	4.8565	4.6936	—	—
Corpus shannon entropy (word) *Synth > Pseudo word diversity*	9.3543	9.9799	—	—
Mean per-document shannon entropy (character) *Pseudo > Synth, significant difference*	4.8449	4.6846	U = 260.00	*p*<0.001
Mean per-document shannon entropy (word) *Synth > Pseudo, significant difference*	8.3075	8.6939	U = 0.00	*p*<0.001
Distributional differences				
JSD (word dist. between corpora) *Moderate divergence*	—	0.3770	—	—
Mean Per-Doc JSD (word-level) vs. combined corpus *Synth more divergent,* *significant difference*	0.2297	0.2618	U = 12.00	*p*<0.001
Linguistic associations				
Corpus average bigram PMI (min freq=3) *Pseudo > Synth word pair associations*	6.9187	5.9833	—	—
Mean per-document bigram PMI *Not statistically significant*	6.4007	6.2604	U = 161.00	*P*=0.2611
Document structure				
Average document length (characters) *Pseudonymized docs much longer*	13,370.15	9,412.25	—	—
Surface & Semantic similarity				
BLEU score (synthetic vs. pseudonymized) *Very low n-gram overlap,* *low surface similarity*	—	4.6179	—	—
BERTScore (synthetic vs. pseudonymized) *Moderate similarity*	—	0.6447	—	—
Classification metrics				
Precision *Semantic/textual discrimination*	—	0.6556	—	—
Recall pseudonymized—F1 *Similarity measure*	—	0.6499	—	—
Classifier AUC (pseudo vs. synthetic) *Perfectly distinguishes* *lower = better for synthetic*	—	1.0000	—	—
Classifier AUPRC (pseudo vs. synthetic) *Perfectly distinguishes (lower = better for synthetic)*	—	1.0000	—	—

Evaluation metrics used—see Table 5, for detailed description—are character and word diversity (Shannon entropy), distributional differences (JSD), linguistic associations (Average Bigram PMI), document length, surface and semantic similarity (BLEU and BERTScore), and the ability of machine learning classifiers to distinguish the two types of documents (AUC and AUPRC). Note, the Mann–Whitney U-test—reporting both statistic and p-value—is used to determine whether observed differences between the corpora are statistically significant. This non-parametric test was chosen because it does not assume a normal distribution, making it suitable for text-derived metrics that often violate this assumption, and thereby provides a robust evaluation of whether differences reflect meaningful distinctions rather than random variation.

The corpus-level Shannon entropy quantifies the diversity and unpredictability of character and word usage within a corpus. For character-level entropy, the pseudonymized corpus (4.8565) exhibited a higher value than the synthetic corpus (4.6936), indicating that texts in the pseudonymized set utilize a greater variety of characters or employ characters in a less predictable manner. This suggests that the process of synthetically generating text may introduce constraints or redundancies at the character level, resulting in reduced diversity.

Conversely, word-level corpus entropy was higher in the synthetic corpus (9.9799) compared to the pseudonymized corpus (9.3543). This reflects a broader or less predictable word usage in the synthetic data, potentially attributable to the generative process introducing new combinations of words or emphasizing novelty. Thus, while synthetic data appears to be less varied at the character level, it is more varied at the word level than the original pseudonymized corpus.

Beyond overall corpus-level entropy, per-document analysis further clarifies differences in diversity and distribution between the pseudonymized and synthetic corpora. Mean per-document Shannon entropy at the character level was higher for the pseudonymized documents (4.8449) than for synthetic ones (4.6846), and this difference was statistically significant (Mann–Whitney U = 260.00, *p* < 0.001). This corroborates the corpus-level finding, indicating that, on an individual document basis, pseudonymized texts are consistently more diverse and less predictable regarding character usage than their synthetic counterparts.

Moreover, mean per-document Shannon entropy at the word level was significantly higher for synthetic documents (8.6939) than for pseudonymized ones (8.3075) (Mann–Whitney U = 0.00, *p* < 0.001). Thus, at the document level, synthetic texts exhibit greater unpredictability and a broader vocabulary than those found in the pseudonymized set, as was the case for corpus-level entropy.

For Average Bigram PMI, which quantifies the strength of association between word pairs by measuring how much more likely two words are to occur together than would be expected by chance, we observed that the synthetic corpus exhibited weaker word pair associations compared to the pseudonymized corpus. Specifically, the corpus-average bigram PMI was lower in the synthetic data (5.98) than in the pseudonymized data (6.92). This indicates that bigrams in synthetic texts are less strongly associated, or less conventional, than those present in the real-world clinical narratives. This finding likely reflects the inherent repetitiveness and predictability of authentic EHRs, in which common phraseology and co-occurring terms are documented repeatedly, thereby increasing the frequency and association strength of certain word pairs. In contrast, synthetically generated texts may introduce more varied or less natural co-occurrences, leading to overall weaker bigram associations.

In contrast to Shannon’s Entropy and average bigram PMI, metrics such as JSD, BLEU score, BERTScore, and classifier performance directly provide a comparative value as output (Table 6). While the synthetic data demonstrates several meaningful similarities to real data, there are also notable shortcomings.

The mean per-document Jensen-Shannon divergence (JSD) of word distributions relative to the combined corpus is higher in synthetic documents (0.2618) than in pseudonymized ones (0.2297), a statistically significant difference (Mann–Whitney U = 12.00, *p* < 0.001). This signifies that individual synthetic documents tend to diverge more from the aggregate corpus word distribution than pseudonymized documents, implying less conformity and potentially greater variation in how topics or vocabulary are expressed in synthetic data.

In surface-level text analysis, an *n-gram* refers to a sequence of *n* consecutive words—such as a bigram (two words) or a trigram (three words)—and comparing n-grams between documents helps reveal how closely their wording and phrasing align. Surface-level text similarity, as measured by the BLEU score (4.62 out of 100), was very low, indicating minimal n-gram overlap between synthetic and pseudonymized texts. This result bears out that the synthetic data are not merely replicating or closely paraphrasing real document phrases but are instead generating genuinely novel text content. While a low BLEU score might be viewed as a negative outcome in tasks requiring close mimicry, in the context of privacy-preserving data synthesis, it is encouraging. It demonstrates that the generative Agent workflow is not merely memorizing or reproducing existing expressions from the source corpus, but creating new, diverse language that reduces risks of information leakage.

Semantic similarity, as assessed by BERTScore, was moderate, with an F1 score of approximately 0.65 (precision: 0.64, recall: 0.66). Thus, although the synthetic data exhibits low surface-level overlap and increased lexical diversity compared to authentic EHR notes, it nonetheless preserves a substantial portion of the underlying clinical meaning and topical content. Such moderation in semantic overlap tells us that the sentences and phrases in the synthetic data are not directly copied or closely matched, word-for-word, with those in the original clinical records (authentic EHR notes). When we look at common sequences of words (n-grams), there is very little overlap between the two sets—meaning the synthetic data displays different combinations of words and sentences, rather than repeating those found in the real notes. In our use case of synthesizing clinical narratives, very high F1, precision, and recall measures would indicate exact copies of original data, whereas our aim was to increase textual diversity by adding real-world samples and clinical practice guidelines as knowledge bases for reasoning.

A machine learning classifier trained to distinguish between synthetic and pseudonymized (real) documents attained perfect discrimination, with both AUC and AUPRC scores of 1.00. This result shows that there are clear, easily learnable feature differences between the two datasets—particularly those reflected in TF-IDF representations. Among these, document length emerged as a primary distinguishing characteristic. Consequently, while the synthetic dataset demonstrates advantages such as content novelty and satisfactory conceptual coverage, its inability to realistically replicate document length results in synthetic documents being consistently and trivially separable from real ones. This highlights the need for improved modeling of document-level properties to enhance the realism and utility of synthetic clinical text. It also provides a clear path forward: simply adjusting the length of synthetic documents to match that of authentic clinical notes, or by deleting redundant information from the authentic clinical notes, should eliminate the primary feature the classifier uses to tell them apart. In doing so, the synthetic texts would likely become much harder for automated classifiers to distinguish from real ones, substantially improving their realism and the utility of the synthetic dataset.

Our corpus-level assessment—drawing on both Figure 4 and Table 6—shows that clinical text derived through GA-assisted SHDG are more predictable at the character level but surpass pseudonymized notes in word-level diversity and unpredictability. This pattern likely stems from artifacts or variability introduced during text generation, with important implications for the realism and utility of synthetic data. Overall, our findings highlight that while synthetic EHRs successfully avoids direct replication and privacy risks by producing novel word combinations, they also exhibit weaker conventional phrase associations and greater divergence from word distributions observed in the real corpus in.

## 5 Discussion

Our present work addresses a persistent barrier in digital health GenAI: the lack of accessible, interoperable, and privacy-preserving datasets that capture the diversity of real-world healthcare documentation (Eigenschink et al., 2023; Murtaza et al., 2023; Hernandez et al., 2025; Ibrahim et al., 2025; Loni et al., 2025). Building on our stepwise strategy for constructing a learning health system (LHS) (van Velzen et al., 2023), we argue that a fully integrated LHS will remain unattainable until these data challenges—especially in nursing and allied health (Tischendorf et al., 2025)—are resolved.

Echoing “*On the Dangers of Stochastic Parrots*” (Bender et al., 2021), we stress that healthcare must ask whether public cloud LLMs can be used in ways that are truly FAIR—Findable, Accessible, Interoperable, and Reusable—while mitigating risks such as high environmental and financial costs, opaque or biased data, and amplification of inequities. This requires curating and documenting high-quality datasets, aligning development with research and stakeholder values, and exploring approaches beyond ever-larger models.

A viable solution is the adoption of compact, fine-tunable SLMs. These small LLM alternatives can be deployed directly within healthcare facilities. Running locally not only reduces reliance on external cloud services but also lowers operational costs, decreases energy demands, and enhances data privacy. SLMs are increasingly capable of powering point-of-care applications—from clinical decision support to patient communication—while ensuring sensitive information remains within institutional boundaries (Schick and Schütze, 2021; Dibia et al., 2024; Garg et al., 2025; Kim et al., 2025; Xie et al., 2025).

By establishing a no-code, protocol for creating GA-assisted SHDG workflows—enabled by rapid prototyping platforms and further enhanced through fully automated, GenAI-assisted co-development—it is achievable to significantly lower the technical threshold for GenAI engagement. Leveraging the DSI stack as a generic blueprint architecture (Figure 1A), our methodological approach illustrates how modular, no-code frameworks can be systematically employed to streamline and democratize the creation of intelligent healthcare workflows.

In particular, the use of GA-assisted workflows to support clinical reasoning for nurse specialists—demonstrated through the “Nandalyse”^11^ tool at the 2025 ASCENDIO conference—represents a major shift in healthcare technology development and adoption in the Netherlands (Kumar, 2023; Jeong, 2025; Tischendorf et al., 2025). These novel GenAI tools enable clinicians and other non-technical users to easily create and customize workflows using simple, modular, drag-and-drop interfaces, significantly lowering the barriers to participation.

A key aspect of our protocol for GA-assisted SHDG workflows is the explicit bridging of the gap between end users—such as clinicians, quality officers, and researchers—and the technical developers responsible for constructing and maintaining AI systems (as detailed in Figure 1B). In healthcare, this chiasm is often perpetuated by differences in language, priorities, and familiarity with digital tools. The explicit integration of human-in-the-loop (Alemohammad et al., 2024) in no-code GA-assisted SHDG workflows not only supports iterative co-development (Li et al., 2023) but also ensures that the system remains transparent and grounded in real-world clinical needs and documentation standards (Chung et al., 2023).

Notably, our methodology fosters closer collaboration between users and developers by providing open-source GitHub repositories (see text footnote 4), which make design choices, workflow logic, and evaluation criteria more transparent and verifiable. However, realizing the full promise of these platforms will require continued investment in user education, ongoing refinement of documentation and support resources, and the cultivation of communities of practice around open-source synthetic data generation.

The successful adoption of privacy preserving GA-assisted SHDG workflows in clinical practice, healthcare professionals requires more than technical proficiency alone (Kumar, 2023; Chew and Ngiam, 2025; Jeong, 2025); they must also possess a comprehensive understanding of key data privacy principles—such as pseudonymization and de-identification—to ensure patient confidentiality is maintained (Drechsler and Haensch, 2024; Rujas et al., 2025). Especially, clinicians should be aware of the inherent limitations of LLMs, including potential risks of bias, hallucination, and model drift, all of which may impact the fidelity and safety of synthetic health data (Alemohammad et al., 2024; Loni et al., 2025). Harnad (2025), notes that LLMs rely on stochastic patterns by capturing statistical regularities rather than achieving genuine semantic understanding, meaning that even fluent and coherent output may be inaccurate, irrelevant or misleading. Shojaee et al. (2025) further caution that the apparent reasoning proficiency of such models can deteriorate markedly as problem complexity increases—an “illusion of thinking” with significant implications for clinical safety. Collectively, these observations reinforce the imperative for multidimensional evaluation frameworks that integrate both surface-level measures (e.g., document length, lexical overlap) and deep-level metrics (e.g., semantic alignment, diversity) to rigorously assess the fidelity and practical utility of synthetic narratives across diverse, high-stakes clinical contexts (Shannon, 1948; Post, 2018; Zhang et al., 2019).

Importantly, working with GenAI in Healthcare mandates a clear understanding of evaluation metrics (Eigenschink et al., 2023; Abdurahman et al., 2025; Ibrahim et al., 2025). Because no single metric can holistically capture the quality of synthetic clinical text, a comprehensive assessment should integrate document- and corpus-level metrics (for overview see Tables 4, 5, respectively): document length and average word count for surface features, entropy for textual diversity (Shannon, 1948), BLEU for lexical overlap (Post, 2018), and BERTScore for semantic alignment (Zhang et al., 2019). Together, these complementary metrics provide a nuanced perspective on both the linguistic and informative value of synthetic narratives. Importantly, high performance on one dimension (e.g., diversity as measured by entropy) does not necessarily translate to strong semantic faithfulness (as measured by BERTScore). This distinction is particularly relevant when evaluating synthetic data for diverse clinical contexts, such as physiotherapy documentation in high-risk or emotionally fraught scenarios (e.g., cardiac, oncology, or orthopedic surgery). Our results (Table 6) reinforce the necessity of using an ensemble of surface and deep metrics to robustly assess the utility and fidelity of synthetic clinical narratives, supporting their appropriate integration into research and practice.

Our work is not without limitations. We identified four main operational and technical challenges that must be addressed to advance GA-assisted SHDG workflows. First, comparative evaluation of on-premises versus cloud-based AI models (Table 1) is needed to optimize trade-offs between data privacy, computational performance, and cost (Schick and Schütze, 2021; Garg et al., 2025; Xie et al., 2025). Parameter tuning—including the adjustment of prompt temperature and chunk size—must be empirically refined to balance realism and diversity in generated outputs (Peeperkorn et al., 2024). Second, while our GA prompts were intentionally crafted to encourage adherence to established clinical practice guidelines to generate clinically relevant outputs, we acknowledge that real-world clinical practice often diverges from these standards. Notably, intentional non-adherence to guidelines has been observed in up to 65% of cases within EHRs, frequently attributable to specific patient factors such as contraindications, comorbidities, or individual preferences (Arts et al., 2016). Therefore, for GA-assisted SHDG workflows to remain practical and reflective of authentic clinical scenarios, addressing this variability is essential. Consequently, our prompt design may have inadvertently constrained the clinical diversity of the synthesized narratives—a limitation that may have been further amplified by the small sample size of our authentic clinical EHR documentation dataset (N = 13 PDF documents). Future SHDG workflows should explicitly address these limitations through testing various prompt engineering techniques to better capture the variability inherent in real-world clinical narratives, or alternatively, by increasing the size of or sample dataset to encompass a broader range of patient presentations, care contexts, and documentation styles and a wider range of intentional non-adherence to guidelines. Implementing these changes—refining prompt-engineering techniques and expanding the dataset—would improve the representativeness of the generated outputs and better align them with the complexities of actual clinical practice. Third, we identified additional prompt engineering issues (Figure 3). These included the inadvertent introduction of gender bias—all synthetic patients turned out to be female—and inconsistent handling of abbreviations—where sample and markdown files contained frequent abbreviations, but synthetic data did not. We also found another prompt engineering issue that resulted in a one-size-fits-all approach in the generated therapy plans and reduced therapy frequency variability. Document length discrepancies, often resulting from repetition in sample or markdown files, also require further exploration to ensure consistent structural fidelity across datasets. Recent research underscores that prompt engineering is not a value-neutral process and that different components of a prompt can vary significantly in their robustness and susceptibility to bias. Mei et al. (2025) provide a comprehensive survey of “context engineering” strategies for large language models, highlighting how the choice, structuring, and sequencing of contextual elements can systematically influence model outputs. Zheng et al. (2025) further demonstrate that individual prompt components—such as instructions, examples, or delimiters—exhibit heterogeneous adversarial robustness, meaning that some parts are more vulnerable to manipulation or unintended bias than others. Related work on priming effects shows that the initial context or examples provided to a model can strongly condition its subsequent responses, amplifying or dampening biases and shaping output diversity (Zhao et al., 2021; Gallegos et al., 2024). Huang et al. (2025) demonstrate that targeted priming can exploit intrinsic weaknesses in large language models, revealing latent vulnerabilities that may not be apparent under standard prompting conditions. Meinke et al. (2024) further reveal that frontier-scale models are capable of sophisticated in-context behaviors, sometimes strategically adapting to earlier cues in ways that can subtly steer reasoning and decision-making. In addition, Gallegos et al. (2024) synthesize evidence that such biases can emerge not only from pre-training data but also from contextual framing and priming during inference. Together, these findings suggest that the gender bias, abbreviation inconsistencies, and homogenized therapy plans observed in our study may stem not only from prompt content but also from the structural composition, priming effects, and resilience of the prompts themselves. This reinforces the need for systematic evaluation and refinement of both prompt components and priming strategies to mitigate bias and improve variability in generated outputs. Fourth, the normalization of clinical text presents a significant methodological challenge, as variations in terminology—for instance, describing the same condition as “sciatica” versus “lumbosacral radicular pain syndrome”—often reflect individual practitioner preferences as well as institutional conventions (Suominen et al., 2013; U.S.-National-Library-of-Medicine, 2024). This semantic variability can be quantitatively monitored using mutual information-based evaluation metrics (see Section 3.4); in our study, we applied the average bigram pointwise mutual information (PMI) metric. However, the robustness of this metric across more diverse datasets and practitioners from different clinical specialties remains to be fully validated. Thus, relying solely on either surface-level metrics or deep semantic measures is insufficient. Instead, a comprehensive evaluation of the faithfulness of SHDG demands the integration of both approaches (Eigenschink et al., 2023; Smolyak et al., 2024; Chen et al., 2025b; Ibrahim et al., 2025; Scherr et al., 2025).

## 6 Conclusion

Based on our findings, we recommend prioritizing the continued development and refinement of GA-assisted SHDG-workflows, ensuring that these toolchains remain accessible, transparent, and customizable for a diverse range of clinical users and researchers through the provision of open-source GitHub repositories (see text footnote 4). Our recommendations align with ongoing efforts by the RUAS Healthcare DataLab to advance innovation in care through collaborative, technology-driven solutions that integrate open, adaptable tools into diverse healthcare contexts.^12^ In parallel, our talent program fosters and equips RUAS students with the skills and expertise required to become proficient data science professionals, thereby strengthening the capacity for data-driven innovation within the Dutch healthcare sector. To maximize impact, future initiatives should emphasize robust user education on essential data privacy concepts—such as pseudonymization and de-identification—and foster a deeper understanding of both the capabilities and limitations of no-code GenAI technologies, particularly regarding risks like bias, hallucination, and model drift. Comprehensive evaluation frameworks should integrate both surface-level and deep semantic metrics to robustly assess the linguistic and clinical fidelity of synthetic narratives, while methodological improvements—including enhanced prompt engineering to address issues of bias, guideline adherence, and structural consistency, as well as adherence to normalization standards—will be essential in capturing the diversity inherent in real-world clinical documentation. Comparative assessments of on-premises versus cloud-based deployments, plus empirical tuning of generative parameters, are also advised to optimize privacy, cyber security, cost, and performance trade-offs for varied healthcare settings. Finally, building active communities of practice and cultivating collaborative, iterative engagement between end-users and developers will be critical to realizing truly FAIR (Mons et al., 2017), AI-ready LHS that can adapt to the complexities and dynamic requirements of modern clinical environments (Huerta et al., 2023; Verhulst et al., 2025).

Our protocolization of privacy-preserving, GA-assisted SHDG workflows underscores the critical importance of maintaining transparency by keeping humans actively involved in the process—a principle known as *human-in-the-loop*. Specifically, we standardized the use of a modular, no-code system in which every step—from designing AI prompts, to providing data inputs, to defining how outputs are applied—can be easily inspected, understood, and refined without programming expertise. This approach enables users to trace how the LLM generates clinical narratives and to make step-by-step improvements over time. In parallel, the integration of an open-source GitHub repository (see text footnote 4), anchored by our modular DSI Stack (Figure 1A), supports the design and deployment of secure, reproducible, and scalable GA-assisted SHDG workflows across both on-premises and public cloud environments. By incorporating Docker-based containerization and API key–secured inference endpoints via Hugging Face Spaces, we ensure controlled access, consistent performance, and community-driven enhancement, while shielding users from underlying infrastructure complexity and maintaining full transparency for healthcare and research applications.

## Data Availability

The sample datasets referenced in this article are not publicly available, as they are proprietary to MediFit Bewegingscentrum Oss and the University of Applied Sciences Rotterdam. At present, approval to use these datasets beyond the university has not been granted. Individuals interested in accessing the sample data are kindly asked to contact Mark van Velzen at m.van.velzen@hr.nl with their request. The original contributions presented in the study—source code, synthetic datasets and prompts—are included in the article/supplementary material. Toolchain implementation details are provided to facilitate reproducibility and to encourage non-AI experts, such as healthcare workers, to confidently explore, adapt, and apply these workflows within their own professional contexts. Further inquiries can be directed to the corresponding author.

## Footnotes

^1^ https://github.com/FlowiseAI/Flowise

^2^ https://github.com/langflow-ai/langflow

^3^ https://www.microsoft.com/en-us/research/project/autogen/

^4^ https://github.com/HR-DataLab-Healthcare/RESEARCH_SUPPORT/blob/main/PROJECTS/Generative_Agent_based_Data-Synthesis/

^5^ https://github.com/HR-DataLab-Healthcare/RESEARCH_SUPPORT/tree/main/PROJECTS/Generative_Agent_based_Data-Synthesis/AGENT-FLOWS

^6^ https://github.com/docker/roadmap

^7^ https://huggingface.co/spaces

^8^ https://docs.flowiseai.com/configuration/deployment/hugging-face

^9^ https://eur-lex.europa.eu/eli/reg/2016/679/oj/eng

^10^ https://digital-strategy.ec.europa.eu/en/policies/regulatory-framework-ai

^11^ https://github.com/HR-DataLab-Healthcare/RESEARCH_SUPPORT/tree/main/PROJECTS/Harnessing%20the%20Power%20of%20Gen-AI%20in%20Research

^12^ https://www.rotterdamuas.com/research/projects-and-publications/innovations-in-care/healthcare-innovation-with-technology/ruas-datalab-healthcare/
